# Visual Search Elicits the Electrophysiological Marker of Visual Working Memory

**DOI:** 10.1371/journal.pone.0008042

**Published:** 2009-11-26

**Authors:** Stephen M. Emrich, Naseem Al-Aidroos, Jay Pratt, Susanne Ferber

**Affiliations:** 1 Department of Psychology, University of Toronto, Toronto, Ontario, Canada; 2 Rotman Research Institute, Baycrest, Toronto, Ontario, Canada; University of Sydney, Australia

## Abstract

**Background:**

Although limited in capacity, visual working memory (VWM) plays an important role in many aspects of visually-guided behavior. Recent experiments have demonstrated an electrophysiological marker of VWM encoding and maintenance, the contralateral delay activity (CDA), which has been shown in multiple tasks that have both explicit and implicit memory demands. Here, we investigate whether the CDA is evident during visual search, a thoroughly-researched task that is a hallmark of visual attention but has no explicit memory requirements.

**Methodology/Principal Findings:**

The results demonstrate that the CDA is present during a lateralized search task, and that it is similar in amplitude to the CDA observed in a change-detection task, but peaks slightly later. The changes in CDA amplitude during search were strongly correlated with VWM capacity, as well as with search efficiency. These results were paralleled by behavioral findings showing a strong correlation between VWM capacity and search efficiency.

**Conclusions/Significance:**

We conclude that the activity observed during visual search was generated by the same neural resources that subserve VWM, and that this activity reflects the maintenance of previously searched distractors.

## Introduction

We use visual working memory (VWM) processes to integrate information across events such as eye blinks and eye movements, where sensory input to the visual system is interrupted [1]–[4]. Studies of VWM have repeatedly demonstrated that the number of detailed representations that can be maintained over short periods of time is limited to three to four visual objects [5], [6]. Memory capacity can be measured using a change-detection task: observers see a display containing a variable number of colored items and are asked to remember as many items as possible. After a brief delay, a probe appears and observers say whether a change occurred. Recent electrophysiological results have revealed that during the delay period of these change-detection tasks, maintaining memory items in a lateralized display is associated with greater negativity over the channels contralateral to the attended side. This difference in amplitude between contralateral and ipsilateral channels, called the contralateral delay activity (CDA), or the sustained posterior contralateral negativity (SPCN) [7], [8], reflects the encoding and maintenance of items in VWM and predicts individual differences in VWM capacity [8]–[11]. As such, the CDA provides a marker of VWM engagement that reflects the involvement of this cognitive resource in a given task. The goal of the present study was to test whether or not the CDA can be recorded during visual search, a task with continuous visual input that is typically considered a hallmark of attentional, rather than VWM, processing [9].

Numerous studies have explored the role of VWM in visual search, resulting in several different and sometimes competing theories about what effect it may have in the search process [10]–[20]. For example, while early studies suggested that there is no mechanism to remember which items have already been searched [21], more recent findings suggest that we use memory to prevent previously selected search items from being re-examined [22]–[24]. This inhibition is thought to be mediated by *spatial* short-term memory, rather than *visual* working memory, as tasks which probe spatial short-term memory interfere with this process [12], [18], whereas tasks that probe VWM do not [25], [26].

Although these behavioral studies indirectly assess the role of VWM under specific conditions, they cannot provide a direct measure of ongoing VWM processing. In contrast, by testing for the presence or absence of the characteristic neural correlate of VWM during search, we can identify whether this cognitive faculty is engaged during a typical visual search task. If the event-related potentials (ERPs) observed in the presence of a continuously presented search display do in fact reflect VWM processing, they should be sensitive to the same manipulations that modulate the CDA during VWM-dependent change detection. That is, the amplitude should be sensitive to the number of items maintained in VWM, and should also reflect individual differences in VWM capacity. Therefore, the CDA may provide a measure of the *number* of items currently held in VWM for tasks without any explicit memory requirement, providing insights into the cognitive processes underlying these tasks. More generally speaking, if VWM is indeed a limiting factor in the performance of a given task (as it is for change-detection tasks), performance on these tasks should be constrained by individual VWM capacity, which will be reflected in the CDA amplitude. This means that the CDA can not only be used to predict behavior in typical tests of visual memory but also be used to predict behavior in an attentional task with continuous visual input.

Overall, the goal of the current study was to determine whether the electrophysiological marker of VWM, the CDA, is present during visual search, and to clarify the relationship between the CDA and visual search performance. The direct comparison between the CDA recorded during a lateralized search task and the CDA recorded during the delay period of a VWM-dependent change-detection task allows for the examination of whether, and to what extent, VWM resources are in fact employed during visual search. The results demonstrate that, for the majority of participants, the visual search and change-detection tasks elicited nearly identical CDAs. Furthermore, VWM capacity predicted both the increase in CDA amplitude during visual search and the behavioral measures of search performance, suggesting that the same neural and cognitive VWM-related resources are used during visual search and change-detection.

## Methods

### Participants

Eighteen volunteers participated in this experiment for partial course credit and/or for monetary remuneration. All participants provided written and informed consent, and all procedures were approved by the University of Toronto Research Ethics Board. Seven participants were excluded from analyses (see below), resulting in a total of 11 participants (ages 19–22, mean age 20.2 years, 4 female). All participants were right-handed and reported normal or corrected-to-normal vision.

### Stimuli and Procedure

Both the visual search and VWM tasks were displayed on a 20-inch CRT monitor located 57 cm from the participant. A resolution of 1600 by 1200 was used, with a display refresh rate of 60-Hz. All stimuli were presented on a grey background (RGB = 128, 128, 128). Participants were encouraged to maintain central fixation during all trials.

#### Visual search task

**Figure 1:**
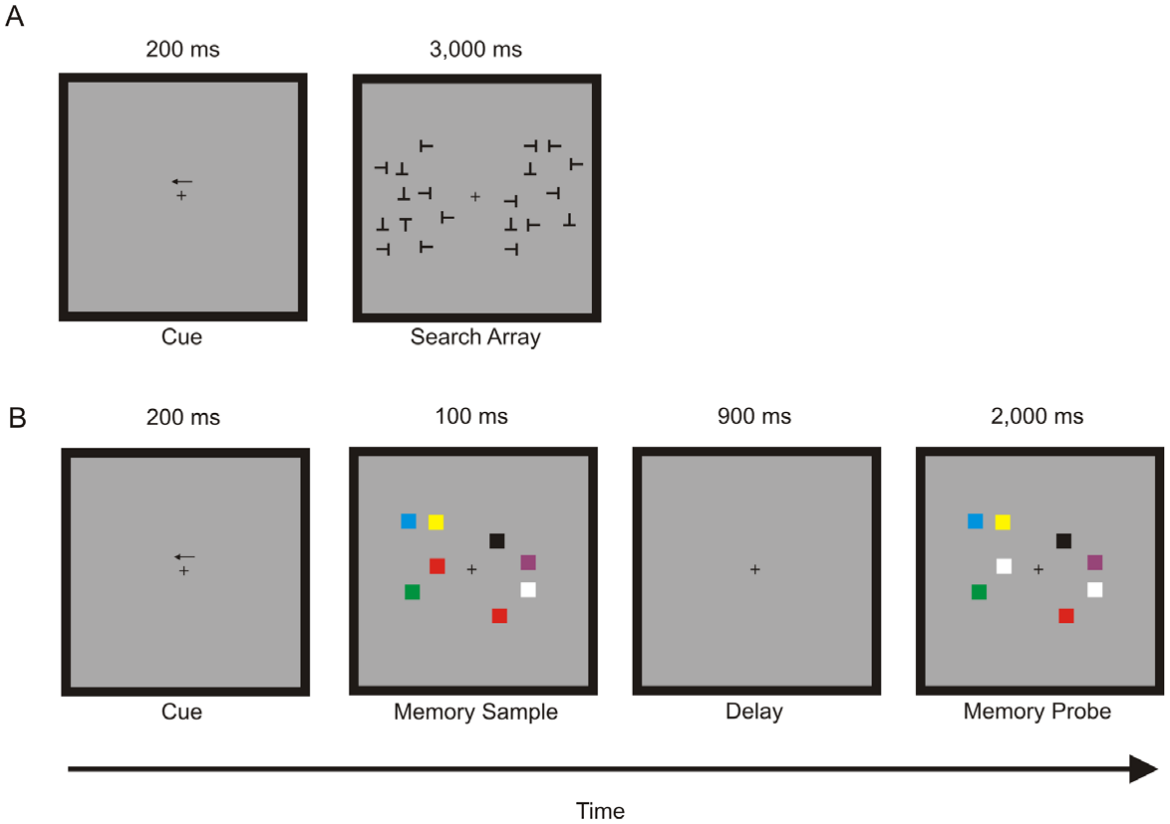
Schematic of experimental trials. (A) Schematic of the visual search trials. An arrow cue indicates which side of the display to attend to, while maintaining fixation on the central crosshair. The cue is immediately followed by the search array consisting of 10 items on each side of the display. Participants were instructed to look for an upright “T” target. (B) Schematic of the change-detection trials. The change-detection task also began with a cue indicating which side of the display to attend to. Participants were asked to remember the colored items on the attended side of the memory sample. Following the 900 ms delay, participants were to indicate whether the memory probe was identical to the memory sample (match trial), or whether one of the items on the attended side changed color (non-match trial).

At the beginning of each trial, a left or right arrow cue was presented, indicating which side of the display participants should attend. The cue was presented 1° of visual angle above a central fixation cross for 200 ms. Immediately after the arrow cue, search items were presented to the left and right of the fixation cross, randomly arranged within a 3.5°×6.5° invisible grid centred 4° horizontally away from the fixation cross. On each trial, ten search items were presented on both the cued and uncued sides (Figure 1A). The search target consisted of an upright “T” shape, and it was never presented on the uncued side. The distractors were “T” shapes rotated 90°, 180° and 270° from vertical. All search stimuli were black (RGB = 0, 0, 0), subtended 0.5° in vertical and horizontal extent, and were presented with a minimum of 0.5° separation. Participants were told to indicate with a keyboard button-press the presence (50%) or absence (50%) of the target on the cued side of the display. The search display was presented for 3,000 ms, and each trial was followed by a 300–400 ms interstimulus interval (ISI). The two factors, Target Presence (present vs. absent) and Cued Location (left vs. right), were completely crossed, resulting in four trial types. Participants performed 100 trials for each trial type, presented randomly, for a total of 400 trials.

Participants always performed the visual search task prior to performing the visual memory task, and were not informed in advance that the second task would test memory performance. Participants were in no way encouraged to utilize a memory strategy while performing the visual search task.

#### Visual working memory task

The procedure used for the visual working memory task was a change-detection task similar to the one used by Vogel and Machizawa [27]. As in the search task, each trial began with a left or right arrow cue that was presented above a central fixation cross for 200 ms. The arrow cue was immediately followed by a memory sample array consisting of four uniquely coloured squares presented in an invisible 1.9°×7.2° grid centred 4.15° to the left and right of the fixation cross (Figure 1B). Seven different colours were used: red (RGB = 255, 0, 0), green (RGB = 35, 177, 77), white (RGB = 255, 255, 255), black (RGB = 0, 0, 0), yellow (RGB = 254, 242, 0), blue (RGB = 77, 110, 243), and purple (RGB = 111, 48, 152). The locations and colours of the memory items were assigned randomly in a given trial, but no one color could appear twice on the same side of the display. This memory sample was presented for 100 ms and then removed from the display. After a 900 ms delay period, the memory test array was presented for 2,000 ms. Subjects were told to indicate with a keyboard button-press whether the memory sample and memory test arrays were identical (50% of trials), or whether one square on the cued side had changed color (50% of trials); item locations never changed, nor did the colors of squares on the uncued side of the display. Each trial was followed by a 300–400 ms ISI. Participants performed 150 trials on each side, for a total of 300 trials.

#### Measuring memory capacity

Visual memory capacity was estimated by applying Cowan's *K-estimate* formula [6] to the behavioral performance on the visual memory task. The formula estimates capacity (*K*) by scaling accuracy, corrected for guessing, by the number of items tested: *K* = *set size* * (*hits*+correct rejections –*1*).

#### Electrophysiological recording and analysis

The electroencephalogram (EEG) was obtained from 64 active Ag/AgCl electrodes (Biosemi ActiveTwo system), digitally recorded at 512 Hz, mounted on an elastic cap using the International 10/20 system. Digital file conversion was completed using PolyRex software [28]. All electrodes were referenced off-line to the average of the left and right mastoids. The horizontal electrooculogram (HEOG), recorded as the difference in activity between electrodes placed lateral to the external canthi, was used to measure horizontal eye movements. The vertical electrooculogram (VEOG), used to detect eye blinks, was recorded from electrodes mounted beneath the left and right eyes and referenced to the frontal electrodes directly above the eyes. A band-pass filter of 0.01–30 Hz was applied offline to the EEG and EOG signals and digitally down-sampled to 250 Hz before averaging. Scalp distributions of the electrical potentials were plotted using BESA software.

Trials that were contaminated with eye blinks (>80 µV VEOG) or large horizontal eye movements greater than 2.0° (>32 µV HEOG) were excluded from analysis [29]. Also, six subjects with trial rejection rates greater than 60% in any single condition were excluded from the sample. The remaining subjects had a mean rejection rate of 42%. The average residual eye-movement for the remaining subjects was less than 0.25° (<4 µV HEOG). One additional subject was removed due to excessive channel noise, reducing the total number of participants to 11. Given the relatively large trial-rejection rate of 42%, a second analysis was performed on the same subjects using a HEOG threshold of 40 µV (2.5°), resulting in an average rejection rate of 27%, with no more than 50% of trials excluded from any one condition. The results of this second analysis were identical to the results presented here.

The ERPs were computed by averaging the EEG from 200 ms prior to the onset of the search display or memory sample display and ending 900 ms post onset. The ERPs were baseline-corrected to the 200 ms prior to the search display or memory sample displays onsets. For all ERPs, analysis was restricted to the 10 posterior channels that demonstrate maximal CDA activity in memory tasks [30]: P5/P6, P7/P8, PO3/PO4, PO7/PO8, and O1/O2.

Ipsilateral and contralateral waveforms were computed separately for the VWM task, and target present and target absent trials in the search task. Laterality was always defined relative to the attended (cued) side for both tasks. The CDA for each condition was computed by subtracting the ipsilateral waveforms from the contralateral waveforms. Latency and peak amplitude measures were obtained using the jackknife method [31]–[34]. The jackknife procedure calculates N grand-average waveforms of N -1 participants, each grand-average excluding a different participant. The peak amplitude and latency values for the local peak amplitude are obtained for each of the N grand-average waveforms, where the local peak was defined as the largest negative voltage peak between 300 and 800 ms post stimulus onset. The latency and peak amplitude values are then submitted to a conventional analysis of variance (ANOVA), where the F-values are adjusted according to the formula
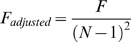

[34].

In addition, to examine the relationship between the increase in CDA amplitude during the search task and VWM capacity, we obtained the change in mean amplitude of the CDA from two 100 ms windows between 300 to 400 ms and 400 to 500 ms in the target absent trials, and correlated these values with VWM capacity obtained in the change-detection task. The comparison of CDA amplitudes between these two early time windows allowed us to assess whether or not VWM capacity was reached quickly during the search task. Specifically, we predicted that participants with low VWM capacity would exhaust their resources with fewer examined search items and as such, their CDA should plateau sooner when compared to high-capacity participants.

We also calculated the amplitude of the N2pc in the target absent search condition for each individual, defined as the maximal negative peak (local minima) between 200 and 300 ms after the onset of the search display. The N2pc reflects attentional selection [35], [36], but is not directly related to VWM capacity and, as such, will allow us to control for the covariance often observed between ERP components and behavioural measures. Analyses were performed on the mean N2pc amplitude, rather than on changes in amplitude over time, as the N2pc is not a sustained ERP. Our method of calculating increases in CDA amplitude over 100 ms windows captures changes in ongoing processes during the first 500 ms, however, the N2pc lasts for only 100 ms and reaches its peak around 50 ms after its onset. Because of these factors, the N2pc is not suitable for examining changes over time but rather reflects a momentary snapshot of attentional selection.

## Results

### Behavioral Results

#### Visual search

Participants correctly reported the presence or absence of the target (upright T) with 61% mean accuracy. The relatively low accuracy of 61% in the search task likely reflects a confluence of factors, including the difficulty of locating targets in the periphery of the display while maintaining eye-fixation on the central fixation cross. In line with previous studies, however, mean reaction times (RTs) in the target present condition (1,301 ms) were significantly faster than in the target absent condition (1,826 ms), t(10) = 13.5, p<.001. Given that the aim of the current study is to examine the electrophysiological correlates relating to the *process* of performing the search, particularly in the first 1,000 ms, electrophysiological analysis was carried out on all trials.

#### Change detection

Mean accuracy at reporting a change to one of the four items in the memory task was 83%, consistent with previous studies using a set size of four items [5]. Mean memory capacity, estimated using Cowan's *K* formula (see Methods) was 2.6 items. Although this estimate is somewhat smaller than the 3–4 item capacity of VWM, this finding is common to studies using a lateralized rather than a central memory array [8].

### Electrophysiological Results

#### Visual search

**Figure 2:**
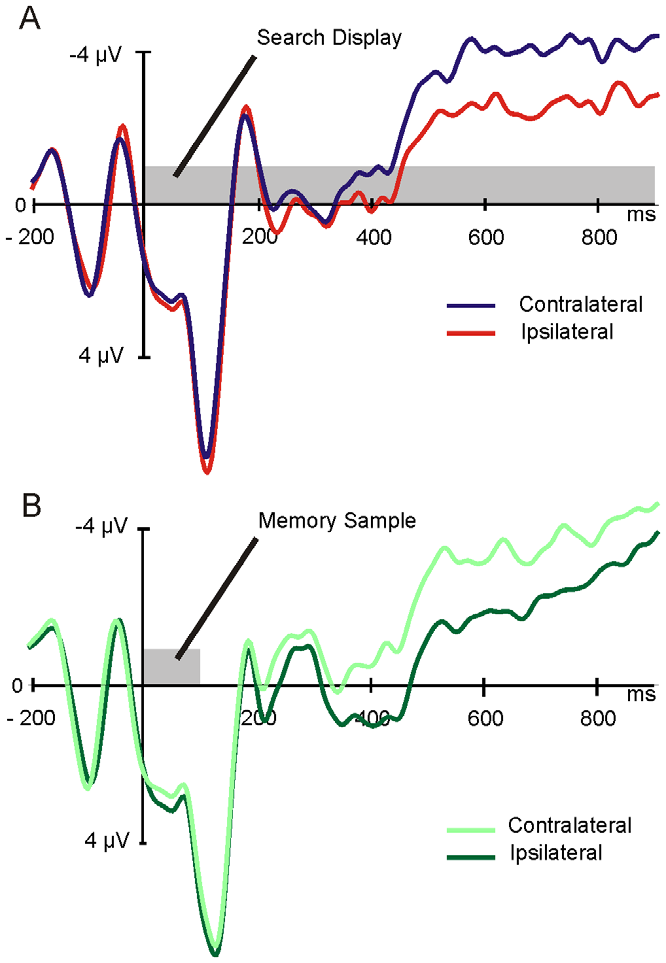
Mean ERP activity. Activity is time-locked to the onset of the search display (A) or the memory sample (B), with the preceding 200 ms used as baseline. For both tasks, activity is collapsed across left and right-cued trials, and plotted separately for channels ipsilateral and contralateral to the cued side. Greater negative activity is evident in contralateral channels relative to ipsilateral channels, beginning around 300 ms, and is sustained throughout the examined window for both the search and change-detection tasks. Negative voltage is plotted upwards by convention.

Activity in posterior channels contralateral to the attended search array was compared to activity in the ipsilateral channels separately for target present and target absent trials, time-locked to the onset of the search array. As can be seen from the resulting ERPs (Figure 2A), contralateral activity diverges from ipsilateral activity approximately 200 ms following the onset of the search array, and then again at approximately 300 ms, persisting thereafter for the entire window examined. The activity from 200–300 ms reflects the *N2pc*, confirming the focus of attention to the contralateral search display [35], [36]. The sustained activity from 300–900 ms, in contrast, resembles the CDA, potentially indicating the use of VWM resources during the search task. Since visual search does not include a delay, we refer to this sustained activity during the visual search task as the contralateral search activity (CSA) whenever it is necessary to distinguish the activity observed during the search task from the CDA normally observed during the *delay* period of a memory task.

#### Change detection

Consistent with previous studies, we found greater negative activity over contralateral channels relative to ipsilateral channels, beginning around 300 ms post stimulus onset (Figure 2B). This negativity is sustained throughout the entire duration of the delay period and reflects the CDA. The change-detection task also elicited an initial *N2pc*, again confirming attention had been shifted to the cued side.

#### Comparing visual search and change detection

**Figure 3:**
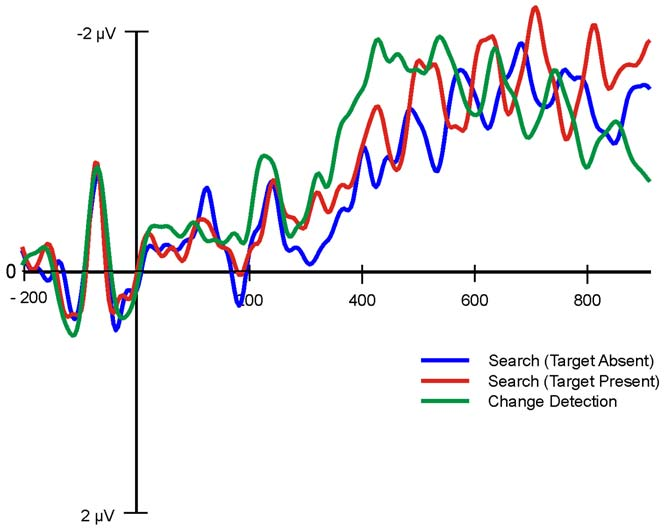
Difference waves for search and change-detection tasks. Difference waves were obtained by subtracting ipsilateral activity from contralateral activity (Figure 2). Activity observed in the change-detection task confirms the presence of the contralateral delay activity (CDA). Similar activity observed in the search (red and blue) trials indicates the presence of the contralateral search activity (CSA). No significant differences in mean or peak amplitude were observed between the CDA and CSA, though the CSA reaches its peak later.

To determine whether the electrophysiological activity recorded during visual search does in fact reflect the use of VWM resources, we compared it to the activity recorded over the same channels during the change-detection task, which is known to depend on VWM resources. Visual inspection of the ERPs recorded during both tasks reveal a striking similarity (Figure 2A/B). That is, the ERPs of both conditions demonstrate large sustained negativity with differences between ipsilateral and contralateral channels emerging about 300 ms post stimulus offset. Difference waves were computed separately for each task by subtracting the ipsilateral activity from the contralateral activity, the resulting difference waves being the CSA (for visual search) and CDA (for change-detection, Figure 3). The mean amplitude of these difference waves from 300–800 ms were compared using a 3-way (present search trials, vs. absent search trials vs. change-detection trials) repeated-measures analysis of variance (ANOVA), revealing that the sustained amplitude did not differ between the three conditions, F(2,20)<1, p = . 7. An equivalent analysis performed on the measure of local peak amplitude (see Methods) also did not reveal any significant differences between the three conditions, F_adjusted_(2,20)<1, p = .78.

To test for differences in the timing of activity, the latencies of the waveforms (see Methods) were compared between the three conditions. This analysis revealed that the CDA elicited in the change-detection task reached its peak amplitude earlier (536 ms) than the CSAs observed in the search conditions (704 ms for target present trials and 680 ms for target absent trials), F_adjusted_(2,20) = 3.08, p<.1 (Table 1). Thus, while the CSA was nearly identical in amplitude to the CDA observed in a four-item change-detection task, indicating that similar resources may have been employed during both tasks, the later onset observed in the CSA indicates that during visual search, resources were employed with a different time-course than during a change-detection task.

**Table 1:** Means and Standard Errors of CDA Amplitudes and Onsets in the Visual Search and Change-Detection Tasks.

	Mean Amplitude (µV) (300–800 ms)		Peak Amplitude (µV) (jackknifed values)		Peak Latency (ms) (jackknifed values)	
Condition	M	SE	M	SE	M	SE
Target Present Search	−1.33	0.40	−2.20	0.06	704	0.5
Target Absent Search	−1.15	0.25	−1.90	0.03	680	0.6
Change Detection	−1.43	0.32	−1.96	0.05	536	9.4

**Figure 4:**
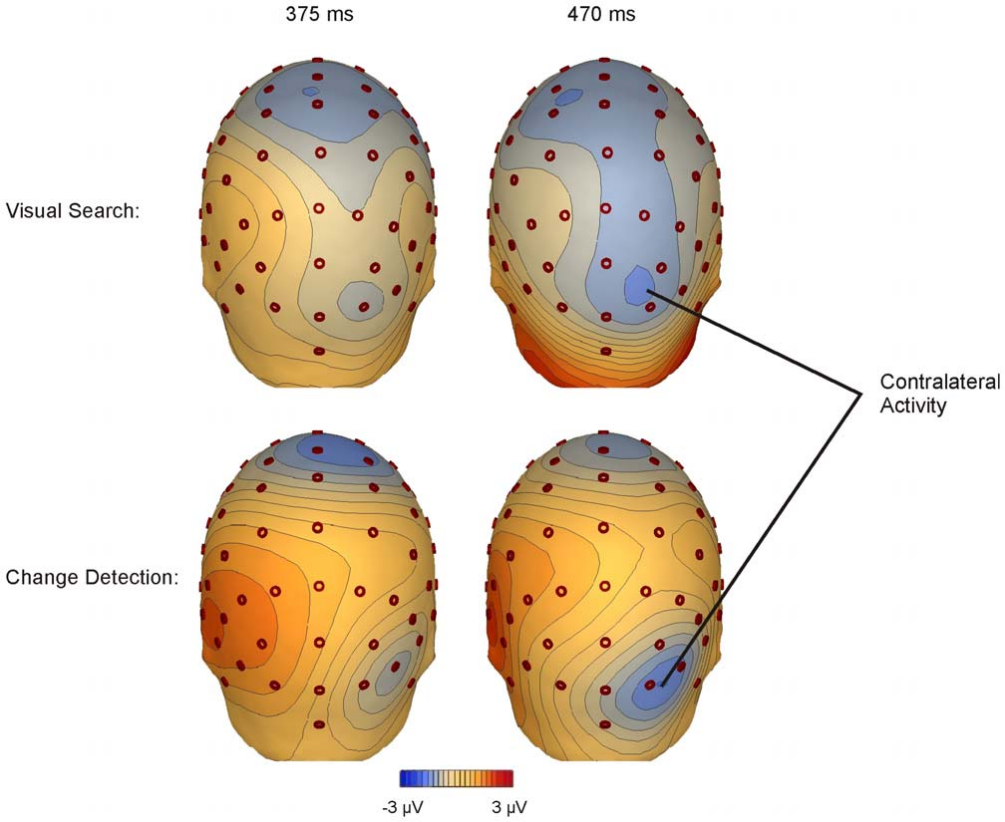
Mean voltage maps for both tasks. For both the search (top) and the change-detection (bottom) tasks, voltage maps are displayed for left-cued trials. Despite large differences in the duration and number of stimuli between the two tasks, similar negative-voltage activity can be observed over contralateral channels, indicating that the same neural resources were recruited during both visual search and change detection.

To further examine the relationship between the CSA and the CDA, scalp distributions are plotted in Figure 4 for attend-left trials in both the target absent search trials (top panel) and the change-detection task (bottom panel). As expected, some differences in the voltage distribution are evident between the two conditions, since only the search task contains a continuously presented visual stimulus; however, it is also evident that both tasks lead to greater negativity over right posterior channels (i.e., contralateral to the left-cued items) at early (375 ms) and late (470 ms) time points. The similar distribution of this posterior activity for the visual search and change-detection tasks provides further support that the CSA reflects engagement of similar resources, namely VWM.

#### What is stored in VWM during search?

The presence of the CSA during visual search provides evidence that VWM resources are utilized while searching for a target. Given that the CDA reflects the number of items encoded and maintained in VWM, the finding that the amplitude of the CSA is not significantly different from the CDA of a change-detection task suggests that a similar number of items were encoded and maintained in both tasks. The later onset of the CSA, however, potentially indicates that the encoding of items into VWM occurs more gradually during the search task than during the change-detection task.

**Figure 5:**
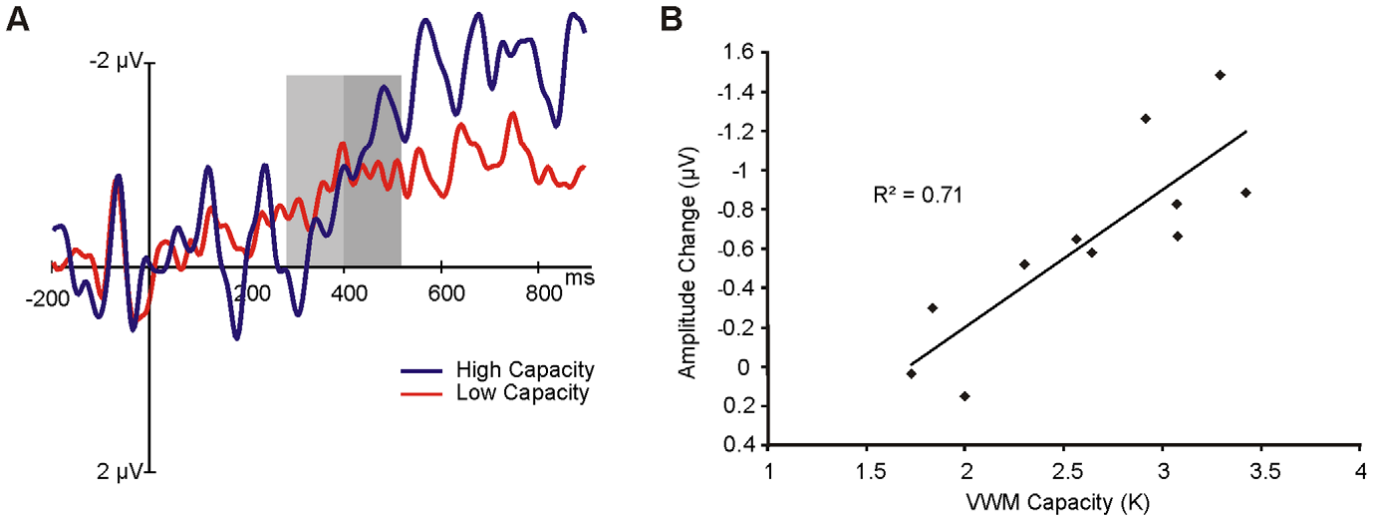
CSA amplitude changes related to VWM capacity. (A) The CSA, plotted separately for high and low-capacity subjects, as measured by the number of items maintained during the change-detection task. The CSA appears to take longer to reach its peak amplitude for the high capacity subjects, potentially indicating that more items were stored in VWM after being searched. The increase in the CSA amplitude from the first 100 ms (light grey) to the next 100 ms (dark grey) window was therefore used as a measure of how sensitive the CSA was to searched items. (B) The correlation between changes in CSA amplitude and VWM capacity, measured in the change-detection task. The change in CSA amplitude was strongly correlated with VWM capacity, indicating that individuals who could store more items in VWM demonstrated a greater increase in CSA amplitude over the course of the search trial, potentially reflecting a greater number of search items encoded in VWM.

If search items were in fact being encoded into VWM gradually, then the time required for the CSA to reach peak amplitude should be related to VWM capacity. Namely, high-capacity individuals should be able to encode and maintain a greater number of search items in VWM than low-capacity individuals, meaning that if both groups encoded items at a similar rate, the CSA in high-capacity individuals may have peaked slightly later. The CSA observed in the target absent trials of the search task is plotted separately for the five high (mean: 3.2) and low (mean: 2.1) capacity subjects in Figure 5A. As can been seen from this figure, the CSA for the low-capacity subjects appears to reach its peak much earlier in the trial relative to the high-capacity subjects. Examining the change in CSA amplitude over the first two 100 ms windows after the onset of the CSA (see methods) demonstrated that the increase in CSA amplitude was significantly correlated with VWM capacity, r = −.84, p = .001 (Figure 5B). That is, those individuals who stored more items in VWM during the change-detection task showed a greater increase in CSA amplitude over the course of the search trial.

#### VWM and visual search efficiency

**Figure 6:**
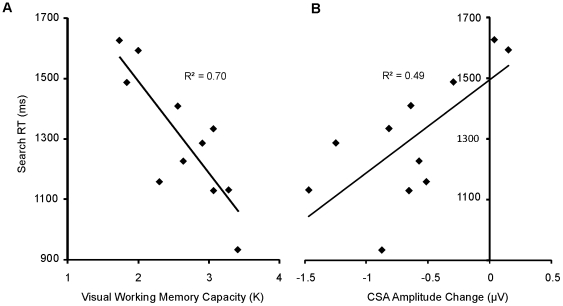
Correlations between search efficiency and measures of VWM. (A) The correlation between VWM capacity and mean search RT. Memory capacity was strongly correlated with search RT. (B) The correlation between the change in the amplitude of the CSA and search RT. Search RT was also significantly correlated with the change in CSA amplitude. Therefore, both independent behavioral measures of memory capacity, and on-line, electrophysiological measures of VWM processing, predict visual search efficiency.

The capacity-related increase in CSA amplitude over the course of the search trial indicates that VWM is engaged during search. Does this mean that by storing more items in VWM, high-capacity individuals will find the target faster? It is possible that if VWM can be used to keep track of some already-visited search items, high-capacity individuals may find the target more quickly than low-capacity individuals, as they will spend less time revisiting those distractors. To test whether visual search efficiency was related to visual working memory capacity, we calculated the correlation between mean RTs on correct-response target present trials with visual memory capacity (Figure 6A). This analysis revealed a strong inverse relationship between memory capacity and search RT, *r* = −.84, *p* = .001. Similarly, the increase in CSA amplitude from 300–500 ms (reflecting the number of search items encoded in memory during the search trial) was significantly correlated with search RT, *r* = .7, *p* = .017 (Figure 6B). Thus, both behavioral and electrophysiological measures of VWM capacity are correlated with search RT.

The observed correlations between memory capacity and search RT indicate that a greater working memory capacity allows search to progress more quickly. It is also possible, however, that this correlation is indicative of a common underlying source of variance that is not specific to working memory, such as general intelligence or task effort. To evaluate this alternate interpretation, we reassessed the correlation between CSA amplitude change and search RT within the context of an additional ERP component that should also correlate with common sources of variance. Specifically, we chose the N2pc amplitude, which relates to attentional selection [35], [36]. Mean N2pc amplitude (M = −1.39, SE = 0.24), however, was not significantly correlated with search RTs, *r* = .41, *p* = .21. Furthermore, when accounting for shared variance observed between the N2pc amplitude and the increase in CSA amplitude, *r* = .40, *p* = .22, the partial correlation between the increase in CSA amplitude and RT remained significant, *r* = .64, *p* = .048. Consequently, the observed relationship between CSA amplitude increase and RTs cannot be attributed to a general source of shared variance, but rather reflects the specific relationship between memory capacity and search RT whereby greater VWM capacity allows for a more efficient visual search.

## Discussion

The aim of the current study was to determine whether the electrophysiological marker of VWM encoding and maintenance is present during visual search, a task that has continuous visual input and virtually no explicit memory demands (other than to remember the target being sought). Our results reveal search-related activity (i.e., the CSA) that is largely indistinguishable from the CDA observed in a four-item, memory-dependent change-detection task. That is, both tasks demonstrate a large, negative, sustained voltage difference between ipsilateral and contralateral channels. Furthermore, the distribution of activity was similar for the two tasks, indicating that the CSA during search and the CDA during a VWM change-detection task likely represent the engagement of the same neural and cognitive resources.

In addition to being identified during change-detection, previous studies have observed activity similar to the CDA during a number of tasks that have both implicit and explicit memory requirements. For example, the CDA has been observed while measuring the deployment of attention during a rapid serial visual presentation (RSVP) stream [7] as well as during masking and cuing tasks [8], [37], [38]. Although these tasks are not explicitly VWM tasks, representations of the stimuli need to be maintained after they have been removed from the display (e.g., after masking or during an RSVP stream). Similarly, one study of multiple object tracking (MOT) observed activity similar to the CDA and found that the amplitude of the observed CDA was sensitive to the number of items tracked, and was related to an individual's tracking capacity [39]. Because the stimuli remain present in the display, no explicit memory representation is required. The nature of the task, however, requires participants to maintain the identities and update the locations of a variable number of stimuli as they move around a display, suggesting perhaps an implicit requirement for VWM resources.

Our results go beyond these previous findings in that they suggest that the neural marker of VWM can be observed even during tasks with continuous, static visual input. Thus, this activity can be used to measure the contribution of VWM to visual search, and potentially other tasks, even when all items remain visible and VWM is not explicitly manipulated. Alternatively, it is possible that the CSA reflects cognitive processing that is unrelated to VWM, such as perceptual or attentional load [40]. We observed three features of the CSA that are consistent with the properties of the CDA established in other studies [27], [30] which argue against this alternative. First, the amplitude of the CSA increases over the course of the search task. This finding suggests search items are encoded and maintained in memory after being examined, resulting in an increase in the CSA amplitude as additional items are visited and uploaded into memory. Thus, the CSA demonstrates sensitivity to the number of items maintained in memory. Second, the amplitude of the CSA is not related to the number of search items, but instead plateaus at the capacity of VWM, consistent with the effects observed during VWM tasks. If the CSA were sensitive only to the number of items or locations examined regardless of capacity limitations, then the CSA would continue to increase in target absent trials until all 10 items had been examined. In contrast, the amplitude of the CSA increased over the course of the search trial, but reached an asymptote long before the entire set of search items could have been examined. Furthermore, the peak amplitude of the CSA did not differ from the peak amplitude of the change-detection CDA, even though the search display had more than double the number of stimuli than the change-detection task, indicating that the CSA reflects a capacity-limit that is consistent with VWM. Third, previous studies have demonstrated that the difference in CDA amplitude across multiple set-sizes in a change-detection task is strongly correlated with individual participants' VWM capacities [27], [30]. Similarly, our results demonstrate that the increase in CSA amplitude from 300–500 ms is significantly correlated with VWM capacity. Therefore, individual differences in the CSA amplitude are predicted by individual differences in VWM capacity. This finding is particularly convincing as it indicates that electrophysiological measures obtained during visual search are predicted by behavioral measures of VWM capacity obtained independently of search. Taken together, these findings indicate that the CSA reflects the same cognitive and neural resources that give rise to the CDA.

It remains possible, however, that both the CDA observed in change-detection and the CSA observed during search are mostly driven by attentional selection and not VWM per se. In other words, the more efficient an individual's selection process, the better their VWM performance and the faster their search. In fact, previous studies have shown that high-VWM capacity individuals select relevant items more efficiently, whereas low-capacity individuals show greater interference from irrelevant items [41]. Recent evidence, however, indicates that the inability to override attentional capture by distractors arises in early attentional selection processes that occur within about 100 ms of the onset of the distractors [42]. Thus, while the two processes are obviously related, individual differences in attentional selection seem to affect what is encoded into VWM [42], [43]), whereas the CDA likely reflects VWM processes related to the maintenance of its contents [41].

As such, our results suggest that VWM resources are employed while performing a visual search task, and that for most individuals, the number of items maintained in VWM during search is similar to that of a change-detection task. Interestingly, our design did not in any way manipulate VWM load during search, and participants were not instructed to use strategies that would elicit VWM activity during the search task; consequently, it would seem that VWM resources were ‘automatically’ recruited to perform the visual search, having potentially important implications for theories of attention.

### The Role of VWM in Search: Evidence from the CSA

As described earlier, we suggest that VWM supports visual search by remembering what search items have already been processed, allowing them to be subsequently ignored and biasing search towards new items.

There are two alternatives to this interpretation. First, the increase in CSA amplitude may be unrelated to the maintenance of search distractors, and instead may reflect other contributions of VWM to search. The relationship between VWM capacity and search RT, however, provides evidence against this alternative. Previous studies have demonstrated how encoding and maintaining distractors in VWM can have a direct effect on search RT [44]. That is, each item that is not stored in memory cannot be removed from the list of candidate targets and may be revisited, resulting in longer RTs [44]. Our finding that search RT is strongly correlated with VWM capacity is consistent with search items being stored in VWM: maintaining more items in VWM decreases the number of items that have to be searched, resulting in an overall decrease in search time.

Second, numerous behavioral studies have indicated that the mnemonic mechanism that prevents previously searched distractors from being revisited [22], [23], [45] depends on a purely *spatial* short-term memory system that is distinct and independent from VWM [25]. According to this hypothesis, the CSA may reflect spatial short-term memory processes. The relationship between the amplitude of the CSA and VWM capacity, however, strongly suggests that VWM plays some role in maintaining and inhibiting search distractors. This conclusion is consistent with the findings that inhibition during visual search may be limited to roughly four items [23], [46], [47] (consistent with the capacity of VWM) and that distractor devaluation in visual search requires VWM [48]. Given the evidence that spatial short-term memory plays a role in the inhibition of previously searched distractors, however, it is likely that both VWM and spatial short-term memory contribute independently to inhibition during visual search, and ultimately to search efficiency. The conjunction of VWM and spatial short-term memory may support inhibition jointly by combining information about both the identities and the locations of old items. In addition, spatial working memory may play a role in prospective memory, guiding attention towards a to-be-selected item [24], indicating that these processes may in fact have distinct roles in search. Future studies are required to resolve the precise effects of these systems on visual search.

Although our study presents the first demonstration relating the CSA to VWM, previous studies have demonstrated CDA-like activity during visual search [49]–[51]. Interestingly, one of these studies demonstrated that the amplitude of this sustained activity was smaller when there was only one potential pop-out target relative to when there were two potential pop-out targets (i.e., when some of the items needed to be searched and rejected) [51]. This finding further demonstrates that the CSA may be modulated only by the number of items searched and rejected, consistent with our suggestion. Other studies have demonstrated that the amplitude of the CDA activity during search can be modulated by motivational factors [52], suggesting that examining the CSA during visual search may prove extremely useful for uncovering and understanding individual differences in cognitive strategies and behavioral performance.

In summary, our visual search task elicited activity contralateral to the attended search array that was indistinguishable in mean and peak amplitude from the CDA observed during a four-item change-detection task, despite large differences in the number of items in the display and length of stimulus presentation. The change in amplitude of this CSA over time was strongly correlated with VWM capacity, suggesting that the activity reflected the same resources employed during VWM tasks. Furthermore, behavioral measures of search performance were strongly correlated with electrophysiological measures of VWM processing observed during the search task (i.e., the CSA), as well as with behavioral measures of VWM performance obtained on an independently performed change-detection task, suggesting that VWM plays an integral role in visual search. This occurred despite the absence of explicit VWM requirements. Consequently, the finding that visual search gives rise to the electrophysiological marker of VWM indicates that the CDA may provide a useful tool for identifying the role of VWM in tasks which have continuously presented stimuli and no explicit memory requirement.
